# Supplementary material for: Understanding stakeholder perspectives on Apgar score, cyanosis and identifying jaundice in ethnic minority neonates
Source: PLoS One. 2024 Oct 15;19(10):e0311977. doi: 10.1371/journal.pone.0311977 (PMC11478820; doi:10.1371/journal.pone.0311977)
Supplement: S1 File — (DOCX) [file pone.0311977.s001.docx]

**S1: Quotations:**

**Understanding Stakeholder Perspectives on Apgar Score, Cyanosis and Identifying Jaundice in Ethnic Minority Infants.**

## Apgar Scores

### Assessment in Practice:

*“I do it quickly, then you hand them over to the paeds and then I say, ‘oh can I just check the Apgar scores if it matches with what I’ve got?”* (OB02 – Black)

*“To be honest, we don’t really do [it], I mean we, if the baby’s fine, you just kind of say it’s 9 and if they’re in trouble, then you kind of sort them out and then, in retrospect, you assign an Apgar score … It’s one of those things you have to do for the notes you know* … *you don’t go through all of this if they don’t need resuscitating*.*”* (NNP08 – White)

*“We don’t look at the colour as such, but we do know what an abnormal colour looks like.”* (MW13 – White)

*“It’s when they come out and you know those ones that they’re not crying immediately. Then everybody stops and really counts the Apgar properly.”* (OB02 – Black)

*“I think I an argument that the Apgar score is not the best method of assessing well-being, you know. And sometimes it’s quite, it’s quite controversial in two or one. Well, the difference between you know, one and none or one and two in the difference with Apgar scores.”* (NNP03 – White)

*“I don’t know what midwives do, whether they look at the soles of the feet or the palms of the hands, or what they do, whether they just think this baby’s fine, they’ll just tick the number 2 on the appearance.”* (NNP08 – White)

*“I would probably argue that if you assess Apgar scoring and the way persons assess it in countries that are probably predominantly of African descent, it’s going to be a completely different. Well, this is what I’m assuming I haven’t assessed the actual data myself, but I would think that it’s going to be completely different compared to, if you assess, if it’s assessed in a country where Caucasian is the majority of patients.”* (NNP09 – Black)

*“I only knew through an antenatal course I did. That they look at skin colour when the baby is born to give a score.”* (PA21 – Other)

*“She [the midwife] did tell me that our skin colour would quite be a little bit um, should I say tough for us to care for and I didn’t really get what she meant by that.”* (PA11 – Black)

*“But when a Black baby’s first born, they’re not always, their pigment is not always fully developed then, so you can see clearly.” (*MW07 – Black)

*“She [3^rd^ child] got brown the quickest out of all my other children. The other two I generally thought they were albinos. So, I was like, I know we come out quite light, but I was just like oh wow and they took a very long time to get darker.”* (PA04 – Black)

### Relevance of the Apgar score to ethnic minority neonates:

*“To decide on the Apgar score of this baby, you can see whether a baby’s colour is, whether baby’s skin is not well oxygenated. But they do talk about whether baby is centrally pink, centrally blue and you can see that whether the baby is darker skinned or not.”* (NNP07 – White)

*“I suppose that under the skin there’s a kind of pink undertone. Yeah, and that would kind of tell me that the baby is like well.”* (HV05 – Black)

*“Pink to me is a bit of an oxymoron, really. It’s like, you don’t get pink Black or Asian babies, do you?”* (MW02 – Black)

*“Certainly, pink is never going to happen in a Black African baby.”* (MW13 – White)

*“You probably would find the average Black staff or brown staff won’t call a baby pink, if they’re Black. They won’t do it because we don’t think they’re pink.”* (MW07 – Black)

“*We do say pink, but we just mean not blue. When we’re saying the baby is pink and well perfused, we say it frequently in babies that are not Caucasian, what we’re saying is the baby is not cyanosed and not anaemic. Basically, it’s what we’re trying to say. Yeah, it’s probably not actually accurate because the baby is not pink.”* (NNP04 – Mixed)

When asked how they might assess ‘pink’ in a Black, Asian or minority ethnic baby one midwife replied: *“I don’t know, that is my honest answer.”* (MW11 – White)

When asked whether pink was relevant to their baby one mother responded: *“No!! No no, no not remotely.”* (PA23 – Black)

*“I think the pink was probably like the first couple of days and then she started to get brown.”* (PA04 – Black)

*“Even a White baby doesn’t come out glowing pink and so I don’t think that’s accurate anyway.”* (MW09 – White)

*“I mean is pink even a word that you describe a White person these days?”* (PA14 – White)

*“I’m not a neonatologist, but in my opinion the thing what you’re sharing [Apgar score] is written for White babies.”* (OB03 – Asian)

*“I kind of knew that there can be a difference in terms of how skin tone views and how because the majority of the assessments are based on the European skin tone that can be detrimental to darker skin tones.”* (PA23 – Black)

*“Oh yeah appearance, colour, look at that … Haaa, pink goodness gracious that’s terrible isn’t it!”* (HV03 – White)

*“I’d forgotten that said completely pink … I mean it’s a bit odd, isn’t it?”* (NNP08 – White)

*“It’s [pink] going to be less obvious in a baby of ethnicity.”* (HV08 – Black)

*“Uhm, my honest answer is I don’t really know and that makes me think about what potentially I’ve done in the past when I’ve been looking after these babies. I imagine if I’ve had no concerns about the baby, I’ve probably scored a 2, but thinking back that probably wasn’t correct because I wasn’t able to say that baby was pink.”* (MW11 – White)

*“I think they coded newborn Black babies and Asian bases incorrectly up by giving them a higher Apgar score when I kind of have argued that it was a lesser score ... so they’ve given an Apgar score of 9 or 10 and I’d be more saying, well, I think it’s more 7 or 8 because of the colour.”* (MW02 – Black)

*“**You get good at things because you see it over and over, so it’s hard. But I think we just have to have different pathways reason to be aware that the Apgar score is not as accurate in persons who are not trained in, you know assessing darker skin baby.”* (NNP09 – Black)

*“We’ve got no system, direct system, in place for Black and Asian babies. It’s just a universal thing, that we assess, you know, … we check the colour as best we can in whatever situation that we’re in. Yeah, yeah. But as I said, sometimes it’s not always accurate.”* (MW02 – Black)

*“She was fine, she came out and screamed and peed all over me and then started feeding within seconds. But I think because again, there’s the slight change in tone, isn’t there. So, she came out almost looking a little bit blue, but I think that’s just because her skin tones a bit darker. So, the pinkish hue was perhaps changed slightly.”* (PA23- Black)

### Suggested changes to the Apgar Score:

*“So, its colour, tone, heart rate, respiration and response to stimulus. Those are the five pieces in the Apgar. So, a baby who’s not oxygenating well, won’t have good tone, won’t be breathing well and so it’s [colour] just one part of that assessment puzzle”* (NNP07 – White)

“It’s not always accurate. And sometimes, unfortunately, we compensate with the other, with the other measurements and with the heart rate, with the breathing, with the tone, with the reflexing.” (MW02 – Black)

*“If my baby still got slow heart rate and not breathing, I don’t really care what the tone is doing.”* (NNP03 – White)

If *“the Black baby was born and it is floppy then the chances are it’s also cyanosed as well.”* (MW03 – Black)

*“You know how to sort of rate it based on the whole picture maybe, so all that baby is crying and alert and sort of showing normal reflexes. So, you’d give it a, you might give it a 10.”* (MW12 – White)

*“I would be looking at the pulse. I’d be looking at their respiratory rate, their tone or attitude in this one, they’re grimace and their heart rate as well. If that was indicated at the time, so that’s how I performed that assessment. I would notice if the baby was very pale, or if the baby was blue or had like a cyanoid appearance to it. But yes, I would be looking at the other parameters of my assessment.”* (MW09 – White)

### **Changed to the terminology of ‘pink all over’:**

*“I think when we talk about being pink, it’s not just the skin it’s looking other uhm, sort of mucous membranes. And that’s where I look for signs of cyanosis because of this issue, but it’s not just the skin that you can see signs of cyanosis”* (NNP04 – Mixed)

*“The peripheries you can use it in Black babies because they, you can actually pick that [up]. And again is very difficult because the peripheries when you don’t have a good circulation can be a bit of a problem.”* (NNP01 – Black)

*“For me it’s pink cheeks like I know when he’s got pink cheeks, he’s like well.”* (PA21 – Other)

*“I guess we’d just convert being completely pink to being well perfused and normal looking colour.”* (NNP08 – White)

*“In terms of telling how well a baby was perfused, I’m not sure that I would feel comfortable knowing how to do that.”* (HV04 – White)

*“The thing is when the baby is first born, you know straight away that that baby hasn’t got good circulation and it’s within a minute that that starts to change. So, if you’re looking properly at your baby, you will know that your baby has changed in that minute, which you should really be looking at.”* (MW04 – Asian)

*“I think that one is looking at the colour of oxygenated blood. You aren’t looking at skin colour. Yeah, well you are looking at skin colour and mucous membranes and fingernails and palms. But I don’t think that the use of the word pink urm is discriminatory.”* (NNP07 – White)

*“It’s going to be difficult to find something that’s going to be 100% objective so it’s almost like kind of the best thing that we have maybe we just have to find ways to modify it?”* (OB02 – Black)

*“I think it will be confusing and I think it will be catering to an unnecessary need … I’m not sure what we can change it to that is going to be accurate. Because it must be accurate.”* (NNP07 – White)

*“I feel the Apgar score is kind of, … it’s archaic now. I think we need to move on to something a bit more modern and up to date. Say you know something that recognises a multicultural society that we currently live in because I feel as I’ve kind of stated before that the Apgar score was brought in for White European babies where you know, we’re far from that now. We’re a bit of a, you know, multicultural society now.”* (MW02 – Black)

*“I think we need to design it so that it takes into account that not all babies are necessarily going to be White … At the moment we aren’t, we just aren’t trained in that way, so you’re not even aware of the alternative ways that you can assess [Black, Asian, and minority ethnic neonates].”* (HV04 – White)

*“I think when we start from the beginning, you know take away the word pink and think of something else to put there more relevant to generic …* *I think probably pale or blue should be there perhaps, but the pink I don’t know.”* (MW07 – Black)

*“I do now that I’ve met you! Because I’ve never thought about it before, which is awful. But I’ve never really paid attention to the fact that it says pink in the assessment and like and that makes it really difficult to assess, you know a coloured baby.”* (MW11 – White)

*“I’ve never really thought about it and that’s sort of, you know, naivete on my part.”* (MW12 – White)

*We quite often will document ‘baby is pink alert with good tone’ and that’s our general kind of documentation about all babies if we’re saying that they’re healthy … but personally I changed that to saying a baby is normal in colour and that with good tone.”* (MW09 – White)

*“Maybe even if they just said good colour other than pink, do you know what I’m saying or uhm, you know something like along the lines of colour acceptable.”* (MW02 – Black)

*“When we’re looking at pink and alert, you know we need to change that. We need to change it to skin tone.”* (MW08 – White)

*“It’s someone in authority that teaches you that way of learning, you accept it as normal. And you might actually feel like it’s not correct, but it’s difficult to challenge.”* (MW04 – Asian)

*“So, I think the “pink and well perfused” statements should be scrapped completely and we change that to, you know, assessing the mucous membranes.”* (NNP09 – Black)

*The term Black, pink and well perfused is pretty much for Caucasian persons and it’s not something that I learned practising in medicine in the Caribbean … We have to comment on this differently because you’re missing a big subset of persons in that statement*.*”* (NNP09 – Black)

*“In my world, I don’t think that appearance means an awful lot, so I I’d quite like to get rid of that.”* (MW13 – White)

*“For years now I’ve realised that the Apgar score is total rubbish. Because and it all depends on a good circulation.”* (OB01 – White)

*“What you do you, you know, [when] you’ve got a baby who’s comes out who doesn’t look good and doesn’t immediately cry? Yeah, you dry them. You put, that you do airway neutral position. You assess them, then you give them breathing support. Then you assess them and then you give them circulation support. So actually, what you do is an ABC assessment and maybe that’s, that’s more important.”* (NNP03- White)

### Better acknowledgement and training of assessment in those from ethnic minority backgrounds

*“I don’t recall having any training or any sort of additional education for babies within that BAME category.”* (MW11 – White)

*“We have to change up the examination, so I think moving from this pathway that ‘All neonates and all persons are examined in the same way’. I think we actually have to reassess that and say, ‘Oh maybe we should be doing things a bit differently for persons of darker skin colour”* (NNP09 – Black)

*“I do think that you can assess. You just need to be taught properly to assess it.”* (MW08 – White)

# Cyanosis:

*“So in in hospital I do remember feeling like his breathing was quite quick and I did call the help button, one of the midwives or healthcare assistance came to check … he was quite mucusy anyway, so it was quite loud so I could hear his breath, so it’s kind of like brought to my attention a bit more.”* (PA21 – Other)

### Detection of cyanosis:

*“I can think of some discussions that I’ve had with neonatal registrars and when I’ve been training with the neonatal teams and the fact is that they haven’t even realized how limiting using colour as a sign of deterioration is, until we’ve had that conversation. And it just goes to show how deep rooted the kind of taught mechanism is and how long it’s going to take to unpick it.”* (MW04 – Asian)

When asked what signs they may look for to see if their child was not getting enough oxygen: *“I assumed they would go slightly blue, but of course that also might be what happens with White babies and I don’t know what happens with mixed or Black babies, so it’s a really good question*.” (PA22 – Mixed)

*“Even dark skinned babies are showing that this skin is well oxygenated because it, because when perfusion isn’t good, you can see that … well perfused skin, it transcends colour.”* (NNP07 – White)

*“More importantly speaking to the parents, people tend to forget and I always say this to my mum’s if you feel that your baby is unwell and you know, you’re kind of a perceived to be a paranoid mum. And I said, you sing it from the rooftops … they know when their babies unwell*.*”* (MW02 – Black)

*“You still have to look for blue, you know … And that is, that’s very important because a Black baby can be blue.”* (MW07 – Black)

*“You can certainly see if they are looking pale or if they’re looking blue because they look darker and they look bluer. There’s maybe less contrast, uhm but you can certainly assess whether they are bluer.”* (NNP07 – White)

*“Black babies don’t look blue.”* (MW03 – Black)

*“What I’m seeing cyanosis looks like in a White skin tone you’re going to miss it in a Black, Asian, you know Asian skin tone.”* (NNP03 – White)

*“I think cyanosis this is one of the things where unless you have lots of experience, it can be very difficult to spot.”* (NNP01 – Black)

*“As a Black baby it is very, very difficult for you to be able to detect if something is wrong with the skin colour because the skin colour does not change.”* (PA17 – Black)

“*I can’t remember any case where a baby has been missed because they’re cyanosed because they are a baby of colour and I don’t find myself teaching saying that UM, look out for babies of colour because you might miss the cyanosis.”* (NNP07 – White)

*“They [Black, Asian or minority ethnic neonates] go, they go blue in a different way.”* (NNP03 – White)

One mother when asked if she though blue was relevant: *“He’s a mixed baby, his skin is more white. So, my baby, yes. But now I’m thinking about my nephews, my family members, probably not, because if you have like a darker complexion identifying blue could be a bit tricky.”* (PA02 – Asian)

*“I think for the Black babies, it might be kind of difficult to identify that … And if he actually developed that, it would have been kind of difficult for me to assess.”* (PA15 – Black)

*“It’s going to actually be the same type of blue colour yeah, because the yellow colour in my child eye was quite similar to that of a White child.”* (PA11 – Black)

*“It tends to look just very dark and lacking in colour so it I would say they probably will never look blue.”* (NNP09 – Black)

*“I would assume it would be quite light to start with, which also will make it difficult to identify. The difficult thing is actually is of course, it’s compared to normal and when your babies a day old, you don’t really know what normal is.”* (PA22 – Mixed)

*“I would say that probably in the UK, I would say it’s almost in a way, it’s hit and miss. In that some babies you know I’ve seen some SHOs show that are very worried because a baby doesn’t pink up … and I go to review the baby and the baby is clinically fine they’re just darker skin … Whereas I’ve seen other cases where … they call me for another reason … and I look at them and I say, actually, this baby is quite blue, so and we put on the sats and it's quite low.”* (NNP09 – Black)

*“Sometimes it’s quite difficult because you might have a child who is severely unwell and because of the dark skin, you might not see the cyanosis there. The lips that are going blue, you might not see that.”* (HV02 – Black)

*“What happens is if people are not confident in detecting it, things you know you could get, you could have a really, really unwell baby that’s been unwell for hours and nobody knows because ‘ohh but the baby’s colour looks fine’”.* (HV08 – Black)

*“I don’t know many people who would be confident enough to say this person cyanosed I don’t need a sats monitor, we would always use that as a sort of a backup.”* (NNP04 – Mixed)

*“It’s not something that I see often because most of the babies that I see they’re at home, they’re well, you know.”* (HV08 – Black)

### **Training needs for detecting cyanosis in ethnic minority neonates:**

*“Considering that we get a yearly update of neonatal resuscitation, BAME is not part of that at all, really reflecting on it. And assessing babies from BAME backgrounds does not get covered at all. It is just a bit of a one stop shop for everybody.”* (MW12 – White)

*“I really don’t think I’ve got a good, you know a good working knowledge of what I should be looking for in terms of skin colour.”* (HV03 – White)

*“We need more training on that to understand what is normal colour for babies, right? … I wouldn’t be able to really describe that to you off the top of my head … Can I say what I think is normal in colour for a Black or brown baby? No, because it’s not something that I’m exposed to.”* (MW09 – White)

*“Learning about what to look for in different skin tones is basic. And it should be taught same way you know if you’re doing hairdressing, I would expect you to know how to do all types of hair.”* (PA04)

*“The answer is that was a really good question. It’s never followed up with an action or any action points. So, when in regards to your question, it’s not very much training that that happens at all, in fact there’s none.”* (MW01 – Mixed)

# Jaundice

*“In the last two weeks I’ve admitted 4 babies with jaundice, 2 Asian, 2 Black babies with jaundice you know, it’s a regular occurrence with me.”* (MW02 – Black)

*“To be honest and I can’t think of any babies with very very, very dark skin that I’ve been concerned about.*” (HV04 – White)

### Detection of Jaundice:

*“I didn’t know that you had to look at the eyes and … the gums … the wee. I didn’t know any of that until I’d actually looked it up myself. Yeah, nobody even in the hospital, no midwife, no support worker, no doctors, no nobody had pointed that out.”* (PA14 – White)

*“I’d probably look at his face in his eyes, his eyes, the whites of his eyes. But I guess then any of his skin I don’t. But then I’m just, I’m guessing here I don’t really know.”* (PA16 – Asian)

*“Always look at the parent if the parent and the child look very similar, you’re probably on around the right road yeah. When the child looks completely different to any of the parents then you think somethings not quite right here … It’s a simple thing, but certainly not fool proof.”* (HV01 – White)

*“I have actually had one recently for a baby who was from a different ethnicity and he appeared to have jaundice and I was pretty certain that he had jaundice, but his parents were quite sallow skinned so you’re not always aware of what’s kind of normal and what is concerning.”* (HV04 – White)

*“Jaundice is one of the issues, because you can never be like 100% sure on OK, this baby looks yellow or it doesn’t. And it has happened, because I’m quite thorough, it has happened that I was like I don’t think so, but let’s do a test and then came into like the highest treatment level kind of thing.”* (MW10 – White) (On a Black baby)

*“I suppose in a way because I don’t feel I’ve got any expertise in skin colour; I would be probably wanting to make sure that the other signs and symptoms that I know to be important I really do explore with the mum. Whereas if again if it was a neonate of White origin and you know, I could see that there was no sign of jaundice from just looking I probably would be less likely to.”* (HV03 – White)

*“We’re seeing babies undressed as well, when you’re looking at the body, you know in the natural light you will see where the tide of jaundice is stopping.”* (HV05 – Black)

*“I would not say it’s at all advisable to do jaundice assessments as a virtual thing.”* (HV01 – White)

### Challenges in detecting jaundice in Black and minority ethnic neonates

*“There was times when it was difficult to tell because of obviously the skin colour because urm, yeah, because of the tone of my kids’ skin tone, it was at times difficult to tell.”* (PA13 – Asian)

*“One of the doctors did once ask me what I thought about his skin colour related to the jaundice, but I was a bit like, “well I don’t really know what he’s supposed to look like, it’s a baby.””* (PA16 – Asian)

*“It can be difficult, you know, especially like the darker the skin the more difficult it can be.”* (HV05 – Black)

*“I know that’s our job in community to identify the babies who are unwell, but I think a lot of these could have been identified earlier.”* (MW02 – Black)

*“I would hate to hear that we are missing more jaundice in Black and ethnic Asian babies possible difficulty in understanding how they may present with jaundice. I mean, I and I don’t know that.”* (HV03 – White)

*“The most severe cases that are re-admitted and tend to be non-White. Umm that’s probably a bit of a broad generalisation, but the ones that need to because they come to the unit and have multiple lights, tend not to be White.”* (NNP03 – White)

*“She [mentor as a student] was an English, you know, she was White. A mature, you know, 40 year midwife. She still failed to identify that jaundice in the Asian baby and that baby needed to be re-admitted. Luckily didn’t have transfusion but it went on the triple phototherapy on the Neonatal unit.”* (MW02 – Black)

*“My partner is a lot darker than I am so possibly because baby came out a mixed colour probably of myself and my partner, so it probably could have been missed for that reason.”* (PA21 – Other)

*“One has essentially to be on the alert and to be more aware of being able to recognise jaundice in a baby who has a darker skin.”* (NNP07 – White)

*“I think you have to be aware that things look different. I think you have to I guess almost not trust your eyes, because you’re actually missing, potentially you can miss jaundice.”* (NNP03 – White)

*“In terms of my routine NIPE examination, even though it says that the jaundice is not part of the examination, is something that I’ve routinely begun to do on the back of this recent risk that we had the baby where it wasn’t picked up, but it was picked up far too late.”* (MW03 – Black)

*“The midwife who came, also wasn’t White, so maybe that’s why she picked it up as well. I can’t remember where she was from, but she had quite similar skin tone to me and maybe that’s why she also noticed it.”* (PA22 – Mixed)

*“I was quite fearing and I was trembling. Yeah, I was like did the doctor do something wrong or did the hospital do something wrong because of my ethnicity?”* (PA11 – Black)

### Addressing Gaps in Jaundice Identification:

*“I would say the jaundice … is a big thing. You’re not taught even now not really taught at university in relation to skin colour and babies on what is normal what’s not normal.”* (MW03 – Black)

*“I have not received any teaching whatsoever in the UK specific to assessing darker skin neonates.”* (NNP09 – Black)

*“I remember at the XXXX local hospital where I work one of the consultant neonatologists specifically telling me don’t look at the skin, look at the sclera and look at the gums.”* (OB03 – Asian)

*“I think the training in terms of identifying cyanosis and jaundice and pallor in children who have different skin colours is really important.”* (NNP04 – Mixed)

“*I didn’t realise it at the time I didn’t. It might, it could have been the skin colour that’s why it was hidden, but I didn’t realise at the time, but I think it was a second appointment or first appointment that the health visitor came out or midwife came out. They did say that he had mild jaundice.”* (PA21 – Other)

*“So, I think the perception of what is a normal colour for an ethnically Chinese sort of Asian person, the perception that it’s normal to be yellow, which of course it isn’t can be life threatening really. And yes, I mean to get admitted to NICU [neonatal intensive care unit] with a bilirubin on the exchange line and then to hear that the midwife when the parents are worried said “oh it’s just your race”, it’s pretty terrifying.”* (NNP08 – White)

*“Listen to the parents if the parents are worried, just do the bloods. Yeah, you know, if you’ve got a parent saying to you “I think my baby jaundice” I mean, they’re probably right.”* (NNP03 – White)

*“You’ve just got to use different strategies and have a lower threshold to check a formal bilirubin otherwise, you run the risk of missing it.”* (NNP04 – Mixed)

*“I have seen babies that hardly look jaundice and they have quite high levels when you test them. And I was lucky because I was in a hospital environment that I could test them and they were lucky also because they were over 24 hours so I could use the transcutaneous machine.”* (MW10 – White)

*“The TCB monitor that we use tends to overestimate in darker coloured neonates …I think the TCB works really well on babies who are lighter skinned, … and when these children come in [to hospital] then it’s much closer to the line. So, I would say I probably see a lot more children who are of darker skin coming in to be assessed for jaundice because the TCB is not working as well.”* (NNP09 – Black)

*“A mother was worried the Billi metre gave a low reading and the midwife did a bilirubin and it was above treatment level, so the baby came in. And Billi Metre had read over a hundred below that.”* (NNP03 – White)

*“Our bilirubinometer, if it’s over 250 you have to do an SBR … I did a bilirubin, it said it was 160. I still wasn’t happy because this baby was bright yellow was OK and they did it [SBr], it was over treatment … it was a good bilirubinometer, it was calibrated. My point is, you know, there is room for error.”* (MW02 – Black)

*“I think that general rule that it’s harder to assess in infants who have darker skin, so do a blood test. So, I think we have a lot of unnecessary blood tests being done.”* (NNP09 – Black)

*“So, he had jaundice when he was born, but not severely enough to have treatment. But of course, his skin color is darker so it’s harder to tell. So, some of the checks we thought you know they went on a little too long because he was fine, he was just darker than a White baby, basically. And that actually happened to my brother’s baby as well.”* (PA22 – Mixed)
