# Supplementary material for: Understanding stakeholder perspectives on Apgar score, cyanosis and identifying jaundice in ethnic minority neonates
Source: PLoS One. 2024 Oct 15;19(10):e0311977. doi: 10.1371/journal.pone.0311977 (PMC11478820; doi:10.1371/journal.pone.0311977)
Supplement: Attachment — Submitted filename: Letter to reviewer.docx [file pone.0311977.s002.docx]

20.09.24

Amy Megan Furness

Manuscript title: Understanding Stakeholder Perspectives on Apgar Score, Cyanosis and Identifying Jaundice in Ethnic Minority Neonates

Dear Members of the Editorial Board,

Thank you for providing feedback on our manuscript Understanding Stakeholder Perspectives on Apgar Score, Cyanosis and Identifying Jaundice in Ethnic Minority Neonates. We appreciate the reviewers' time and effort and have carefully considered each comment. Below, we provide a point-by-point response to address the suggestions and concerns raised.

Response to reviewers:

1. Please ensure that your manuscript meets PLOS ONE's style requirements, including those for file naming. The PLOS ONE style templates can be found at

The manuscript had now been changed to meet the PLOS ONE's style requirements.

2. “This research was commissioned by the NHS Race and Health Observatory.”

The research was commissioned by the NHS Race and Health Observatory. The funders had no role in data collection and analysis, decision to publish, or preparation of the manuscript.

3. We noted in your submission details that a portion of your manuscript may have been presented or published elsewhere.

This work has previously been published on the NHS Race and Health Observatory, on their website. The study has not been subjected to academic peer review from previous publishing. The work was shared in a full report format without undergoing formal peer review.

Given that this version has not been subject to the rigorous peer-review process that PLOS ONE offers, we believe that submitting the work to PLOS ONE does not constitute dual publication. Instead, it provides an opportunity for these findings to be critically evaluated, validated, and made accessible to a broader academic audience through a peer-reviewed journal, ensuring their relevance and impact within the scientific community.

4. We note that your Data Availability Statement is currently as follows: [All relevant data are within the manuscript and its Supporting Information files.] . Authors must share the “minimal data set” for their submission. PLOS defines the minimal data set to consist of the data required to replicate all study findings reported in the article, as well as related metadata and methods (<https://journals.plos.org/plosone/s/data-availability#loc-minimal-data-set-definition>).

All relevant data are within the manuscript and its Supporting Information files.

5. Your ethics statement should only appear in the Methods section of your manuscript. If your ethics statement is written in any section besides the Methods, please move it to the Methods section and delete it from any other section. Please ensure that your ethics statement is included in your manuscript, as the ethics statement entered into the online submission form will not be published alongside your manuscript.

The ethics statement has been moved to methods section.

6. Please include captions for your Supporting Information files at the end of your manuscript, and update any in-text citations to match accordingly. Please see our Supporting Information guidelines for more information: [http://journals.plos.org/plosone/s/supporting-information](https://eur02.safelinks.protection.outlook.com/?url=http%3A%2F%2Fc05y1x9s.r.us-east-2.awstrack.me%2FL0%2Fhttp%3A%252F%252Fjournals.plos.org%252Fplosone%252Fs%252Fsupporting-information%2F1%2F010f019207958702-c7640dc4-5f65-4037-92c7-e4ccbd4d61ed-000000%2FvtwQr4CJb9xCVPEcF5is9JM3eaQ%3D177&data=05%7C02%7CA.Furness%40shu.ac.uk%7C2ca63d472ea647b4a18808dcd858ad2a%7C8968f6a1ac13472fb899f7316e439f43%7C0%7C0%7C638623122571837871%7CUnknown%7CTWFpbGZsb3d8eyJWIjoiMC4wLjAwMDAiLCJQIjoiV2luMzIiLCJBTiI6Ik1haWwiLCJXVCI6Mn0%3D%7C0%7C%7C%7C&sdata=Zk6VelWJs3sYtY63FOXOHOYwIMQkp8sid6N1wpQAF3k%3D&reserved=0)

Captions have been added to the end of the manuscript.

7. Please review your reference list to ensure that it is complete and correct. If you have cited papers that have been retracted, please include the rationale for doing so in the manuscript text, or remove these references and replace them with relevant current references. Any changes to the reference list should be mentioned in the rebuttal letter that accompanies your revised manuscript. If you need to cite a retracted article, indicate the article’s retracted status in the References list and also include a citation and full reference for the retraction notice.

No changes have to be made to the reference list.

We hope the revisions adequately address the reviewers’ concerns. Thank you again for your feedback and consideration.

**Sincerely,**

Amy Furness

Sheffield Hallam University
